# Surgical Treatment of Peri-Implantitis Using a Combined Nd: YAG and Er: YAG Laser Approach: Investigation of Clinical and Bone Loss Biomarkers

**DOI:** 10.3390/dj11030061

**Published:** 2023-02-24

**Authors:** Ioannis Fragkioudakis, Antonios Kallis, Evangelia Kesidou, Olympia Damianidou, Dimitra Sakellari, Ioannis Vouros

**Affiliations:** 1 Department of Preventive Dentistry Periodontology and Implant Biology, Dental Faculty, School of Health Sciences, Aristotle University of Thessaloniki, 54124 Thessaloniki, Greece; 2 Department of Periodontology, School of Dentistry, National and Kapodistrian University of Athens, Thivon 2 Str., Goudi, 11527 Athens, Greece; 3 2nd Department of Neurology, AHEPA University Hospital, Aristotle University of Thessaloniki, 54124 Thessaloniki, Greece

**Keywords:** biomarker, laser, peri-implantitis, surgery, treatment

## Abstract

The current study aimed to investigate the effect of the combined Nd-Er: YAG laser on the surgical treatment of peri-implantitis by evaluating clinical markers and biomarkers of bone loss (RANKL/OPG). Twenty (20) patients having at least 1 implant diagnosed with peri-implantitis were randomly assigned to two groups for surgical treatment. In the test group (n = 10), Er: YAG laser was used for granulation tissue removal and implant surface decontamination, while Nd: YAG laser was employed for deep tissue decontamination and biomodulation. In the control group (n = 10), an access flap was applied, and mechanical instrumentation of the implant surface was performed by using titanium curettes. The following clinical parameters were evaluated at baseline and six months after treatment: Full-mouth Plaque Score (FMPS), Probing Pocket Depth (PPD), Probing Attachment Levels (PAL), recession (REC), and Bleeding on probing (BoP). Peri-implant crevicular fluid (PICF) was collected at baseline and six months for the evaluation of soluble RANKL and OPG utilizing enzyme-linked immunosorbent assay (ELISA). Baseline clinical values were similar for both groups, with no statistical differences between them. The study results indicated statistically significant improvements in the clinical parameters during the 6-month observation period in both groups. More specifically, PPD, PAL, and REC were improved in the test and control groups with no differences in the between-groups comparisons. However, a greater reduction in the BoP-positive sites was noted for the laser group (Mean change 22.05 ± 33.92 vs. 55.00 ± 30.48, *p* = 0.037). The baseline and six-month comparisons of sRANKL and OPG revealed no statistically significant differences between the two groups. The combined Nd: YAG—Er: YAG laser surgical therapy of peri-implantitis seemed to lead to more favorable improvements in regard to bleeding on probing six months after treatment compared to the conventional mechanical decontamination of the implant surface. None of the methods was found superior in the modification of bone loss biomarkers (RANKL, OPG) six months after treatment.

## 1. Introduction

The most common biological complications regarding dental implants are Peri-implant mucositis and Peri-implantitis with peri-implantitis being described as an inflammatory lesion affecting the peri-implant mucosa characterized by gradual bone loss [1]. Several risk factors have been proposed as potential co-drivers in this entity, including a history of periodontitis and poor plaque control [2].

Treatment of peri-implant lesions typically includes mechanical debridement of biofilm and calculus [3]. This is often achieved through professional care and/or by the patients’ effective oral hygiene procedures. Several protocols have been suggested for the non-surgical or surgical treatment of peri-implantitis. They usually involve mechanical debridement of the implant surface using curettes, ultrasonic devices, air-abrasive devices, or lasers, alone or combined with some sort of chemicals mainly based on local antibiotics or antiseptics such as chlorhexidine [4]. In the majority of advanced peri-implantitis lesions, the mechanical non-surgical treatment alone was ineffective, and a surgical approach is suggested in such cases [5]. The most common surgical techniques for surgical treatment are, access flap surgery, the apically positioned flap with or without ostectomy/osteoplasty, and regenerative techniques [6]. Each of these methods has specific indications depending on the severity of the situation and the morphology of the bone [6,7]. Regenerative methods are recommended in the presence of a crater, defect, or three-wall defect, and autogenous bone or bone replacement can be used to achieve bone fill [8]. In general, if peri-implantitis is already established, the proposed treatments can still be recognized as empirical. From the existing evidence, it seems that no method of decontamination is superior compared to others [8].

Lasers have been utilized in the treatment of periodontitis or peri-implantitis since the early 2000s. Laser devices are distinguished depending on the medium used to propagate the radiation in excimer, solid-state, gas, and diode lasers, each with different properties [9]. So, depending on the properties and the wavelength, each Laser category interacts differently with the tissues [10]. In terms of peri-implant therapy, Er: YAG laser has shown promising results with minimum damage on the implant surface [9]. Nd: YAG laser on the other hand, though seems to cause melting on the titanium surface it is considered highly bacteriostatic, especially in deep tissue decontamination due to its specific wavelength [9]. Studies on laser treatment in peri-implantitis have shown promising results. Deppe and co-workers using CO_2_ laser in combination with bone augmentation indicated substantial bone gain compared to the control group [11]. Studies using Nd; YAG laser are limited concerning the fact that it may provoke damage on the implant surface due to its high permeability [12,13]. The combination of Nd: YAG and Er: YAG laser has not been used in the treatment of peri-implantitis yet, though promising results have been found in the case of periodontitis [14].

Peri-implantitis is defined as a destructive inflammatory disease leading to gradual bone loss; thus, several enzymes, cytokines, and proteins that regulate the pathogenetic process have been investigated as potential predictors of disease activity. In this context, factors such as interleukin-1b (Il-b) and tumor necrosis-a (TNFa) have been detected in higher concentrations in diseased compared to healthy implants [15]. The discovery of the receptor activator of nuclear factor-kB (RANK)/RANK Ligand (RANKL)/osteoprotegerin (OPG) pathway contributed to the understanding of how bone formation and resorption were processed and regulated [16]. RANKL and OPG are members of the tumor necrosis factor (TNF), and binding to receptor activator of NF-kB (RANK) not only regulates osteoclast formation, activation, and survival in normal bone modeling and remodeling but also several other pathologic conditions characterized by increased bone turnover [17]. More specifically, the attachment of RANK to its ligand (sRANKL) triggers the fusion of the pro-osteoclast leading to differentiation into mature osteoclasts, thus activating bone resorption [16,18]. OPG can block this process by antagonizing the binding to RANK, thus disabling osteoclastogenesis in favor of bone formation. Rakic and colleagues detected soluble RANKL at higher concentrations in PICF from peri-implantitis sites than in PICF from healthy implant sites [19]. RANKL and OPG have been recognized as potential biomarkers of peri-implantitis in several studies [19,20,21].Though a lot amount of effort has been given in the study of lasers in peri-implantitis, the behavior of bone loss biomarkers after laser treatment has not been studied yet. In addition, this is the first effort to address the results of a combined laser treatment in peri-implant disease. 

Thus, the current study aimed to investigate whether the adjunctive combined Nd: YAG and Er: YAG laser on the surgical treatment of peri-implantitis could enhance the results in clinical parameters and biomarkers of bone loss (RANKL/OPG), compared to mechanical decontamination.

## 2. Materials & Methods

### 2.1. Study Design—Participant Recruitment

The study was designed as a prospective, randomized, controlled trial of a 6-month duration. Participants were recruited from the Department of Periodontology and Implant Biology of the Dental Faculty, School of Health Sciences, Aristotle University of Thessaloniki. Patients participating in this study should have at least one implant diagnosed with peri-implantitis according to the 2017 world Workshop on Periodontology definition [1]. 

The two groups were the control group (C group), where surgical peri-implantitis therapy was performed with the use of an access flap and mechanical instrumentation of the contaminated implant surface. In the test group (Laser group), surgical treatment was carried out with the conjunctional use of laser irradiation of the contaminated implant surface. 

In order to assess peri-implant osseous defect depth, a radiographic examination was performed at baseline. The inclusion criteria for patients to participate in the study, indicating the existence of peri-implantitis are based on the most recent case definition of peri-implantitis, i.e.,:

- Probing depths of ≥6 mm,
- Bone levels ≥ 3 mm apical of the most coronal portion of the intraosseous part of the implant, and
- the simultaneous presence of bleeding on probing and/or suppuration
- Implants should be loaded for >12 months.

As exclusion criteria were set:

- A severe systematic disease of patients by which a surgical procedure cannot be performed (e.g., bleeding disorders, uncontrolled diabetes mellitus, etc.),
- treatment of peri-implantitis within the previous 12 months,
- antibiotic intake in the last three months before treatment and
- prosthetic loading of implants > 12 months.
- In addition, peri-implantitis lesions indicated for regeneration (3-walled, craters were excluded from the study and were treated accordingly).

The assignment of patients into the two groups of the study was succeeded by a randomization process with the use of computer software (www.randomizer.org, accessed on 17 October 2021). In patients with more than one implant having peri-implantitis, a second randomization procedure with the same procedure determined which implant would be included. The randomization results were placed in opaque envelopes accessible only by the supervisor (I.V.) and given to the treating investigator for each patient, just prior to therapeutic intervention. The present research study was approved by the Ethics Committee of the School of Dentistry. Protocol No. 83/15-06-2020. All participants received written information regarding the aim and the procedures of the study, and afterwards, they were asked to sign their consent. The randomized controlled trial (RCT) registration number is NCT05733234.

### 2.2. Sample Size Calculation

Sample size calculation was performed on a patient-based analysis in order to detect a clinically significant difference in probing pocket depth (PPD) of 1 mm ± 0.75 mm SD between the two groups (control and laser). Type I error was set at 0.05 level and power at 0.80. The minimum required sample size was calculated to be ten patients per group. In addition, in order to detect a clinically significant difference in PPD of 1 mm ± 1 mm SD between the two time periods, within the group, with 0.05 type I error and 0.80 power, the minimum required sample size was estimated to be ten patients per group too.

### 2.3. Treatment Timeline

**Figure 1:**
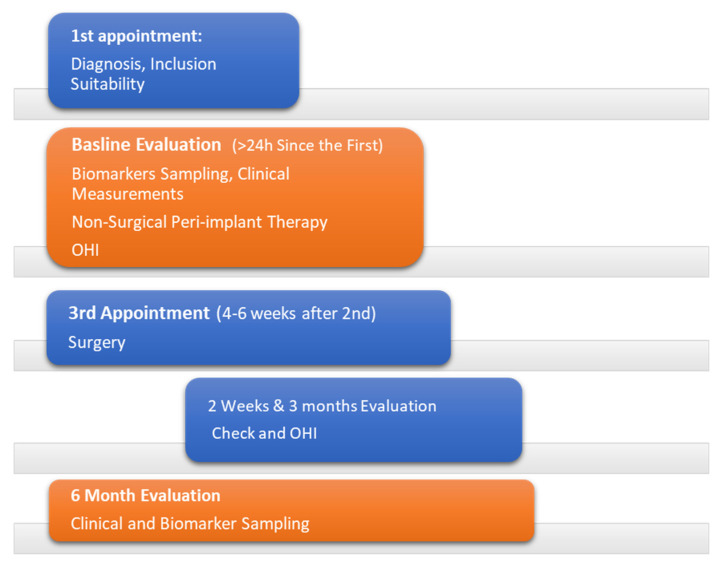
Treatment Timeline.

After an initial clinical and radiographic examination, patients were screened, and accordingly, suitability for enrollment in the study was confirmed. At the baseline examination, clinical measurements, and peri-implant crevicular fluid (PICF) collection for biomarker sampling were performed. The PICF was retrieved prior to probing and at least 24 h after the initial examination to avoid contamination with blood. At the baseline session, after the initial assessment, patients received oral hygiene indications and non-surgical therapy of peri-implant defects in terms of scaling and root planing with titanium curettes and the adjunctive use of chlorhexidine 0.20% irrigation of the peri-implant sulcus. Mechanical debridement using ultrasonics and hand instruments was also performed on the whole dentition prior to surgery. Four-six weeks after the non-surgical treatment the surgery was scheduled. Each patient was followed up for two weeks, three months, and six months post-surgically, and an oral hygiene reinforcement was performed. Clinical measurements and biomarker evaluations were conducted at baseline and six months post-surgically (Figure 1).

### 2.4. Clinical Periodontal Measurements

The following clinical parameters were recorded at baseline and six months after treatment: Full-mouth plaque score (FMPS), percentage of sites positive on bleeding on probing (BoP), probing pocket depth (PPD), gingival recession (REC), and probing attachment levels (PAL).

- BoP: presence (+) or absence (−) of bleeding in percentage (%) 30 s after probe insertion in the peri-implant pocket.
- FMPS: Full-Mouth plaque score.
- PPD: the distance from the mucosa margin to the bottom of the peri-implant pocket.
- REC: the distance from the prosthetic crown shoulder to the mucosa margin.
- PAL: the distance from the prosthetic crown shoulder to the bottom of the sulcus or the peri-implant pocket.

All measurements were recorded at six sites per implant with a 15-mm scale periodontal probe and graded per 1 mm (Hu-friedy^®^ CP-12). The three deepest sites at each implant were included in the statistical analysis for PPD and PAL assessment. All the examinations were performed by the same examiner (I.F.). Intra-examiner reproducibility was calculated during two calibration sessions. Intra-examiner agreement was assessed with an intraclass correlation coefficient (ICC) and showed an agreement of 0.93 (95% CI: 0.89 to 0.96).

### 2.5. Surgical Procedures

Local anesthesia was applied, and each patient used a mouthwash solution of 0.20 % chlorhexidine for presurgical mouth disinfection. After an internal bevel incision, full-thickness flaps were raised to access the peri-implant osseous defects.

**Figure 2:**
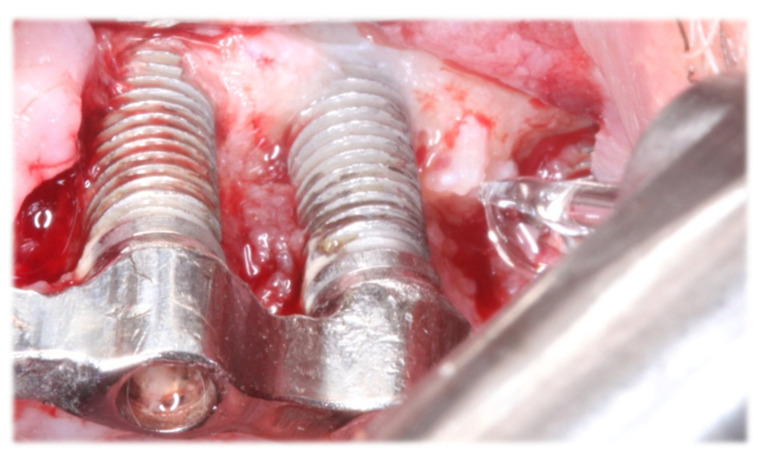
Er: YAG Laser on the Implant Surface.

- Control Group (Group C): removal of granulation tissue and mechanical instrumentation of the implant surface with the use of titanium implant scalers (@Hu-Friedy, IMPLG1/2T) were performed. The instrumentation was followed by thorough cleansing of the implant surface using sterilized gauze soaked in chlorhexidine 0,2% solution.
- Test Group (Group L): In the test group, Nd: YAG laser, 1064 nm (Fotona, Light Walker AT, Ljubljana, Slovenia) combined with Er: YAG laser, 2940 nm (Fotona, Light Walker AT, Ljubljana, Slovenia) was used. Initially, granulation tissue removal was performed by utilizing Er: YAG laser 160 mJ, 10 Hz, LP, 1.3 mm cylindrical tip, handpiece H14-C, W/A:6/4, 30 mL per minute. Additionally, decontamination of the implant surface was performed with Er: YAG laser 2940 nm, QSP mode, 45 mJ, 20 Hz, non-contact, handpiece H02-C, W/A: 6/4 (Figure 2). Bleeding spots and bone disinfection were created with the application of Εr: YAG laser 160 mJ, 15 Hz, non-contact, H02-C handpiece, W/A: 6/4 (Fotona, Light Walker AT, Ljubljana, Slovenia). Following that, the Nd: YAG laser 1.5 W, 15 Hz, MSP, non-contact R21-C3 handpiece, 300 μm fiber was applied for deep tissue decontamination and microbial load reduction. Care was taken for the fiber not to aim at the implant surface. Finally, Nd: YAG laser 0.5 W, 10 Hz, VLP, non-contact R21-C3 handpiece, 300 μm fiber (Fotona, Light Walker AT, Ljubljana, Slovenia) in low level was applied after suturing for photobiomodulation. All laser settings were based on the Lasers physical properties and interaction with tissues and implant surfaces. The protocol was planned in cooperation with the laser company considering previous studies using Er: YAG lasers in peri-implantitis [22,23]. During the laser radiation, both the dental staff and the patients were wearing protective glasses.

In both the control and laser group, osteoplasty was performed when needed for better flap adaptation.

The endpoint of debridement for each treatment was determined by macroscopic inspection of the defect site. After suturing, post-surgical instructions, and analgesics (ibuprofen 400 mg three times a day for four days) were administered to the patients. The sutures were removed about 14 days after surgery, and post-surgical instructions were given to all patients. These included a chlorhexidine 0.12% mouth rinse twice a day for two weeks, for the control group only, and careful tooth brushing with a soft toothbrush so that the sutured area was efficiently cleaned but not traumatized for both groups.

### 2.6. Biomarker’s Collection and Evaluation

Peri-implant crevicular fluid (PICF) and samples were collected from one implant site of each individual participating in the study. The specimens were retrieved at least 24 h after the initial clinical examination to avoid contamination with blood from peri-implant sites demonstrating the deepest probing depth. The samples were retrieved using the filter paper technique [24]. In brief, the sampling sites were isolated, air dried, and isolated with cotton rolls supra-gingival biofilm was gently removed, and then a fine, sterile paper strip was inserted into the peri-implant sulcus/pocket until mild resistance was felt and left in place for 30 s. Strips that were visually contaminated with blood or saliva were discarded. The obtained sampled fluid volume in the strip was measured by calculating the resorbed PICF volume per 30 s, and the paper strips were inserted in micro-centrifuge plastic tubes. The obtained samples were stored at −80 °C until being processed for biochemical analysis by enzyme-linked immunosorbent assays (ELISA).

Before the analysis, the samples were diluted with phosphate buffer solution (PBS), centrifuged for 20 min at 4000 g to separate the cells and debris, and then the paper strips were removed. The obtained samples were stored at 20 °C until being processed for biochemical analysis. Commercially available ELISA kits were used to measure the concentrations of the bone biomarkers: sRANKL and OPG, from the PICF (Biovendor sRANKL (total) Human ELISA (Osteoprotegerin Ligand, OPG (total) Human Osteoprotegerin, Biovendor—Labolatorni medicina a.s—Czech Republic). The minimal detection limits were: sRANKL (0.4 pmol/L), and OPG (0.03 pmol/L). The ELISA in brief, the monoclonal antibodies specific for these biomarkers were pre-coated into a micro-plate. After washing off unbound substances, an enzyme-linked polyclonal antibody specific to the biomarker was added to each well. The plate was incubated at room temperature for 1 h and the wells were re-washed. After one hour of incubation, a stop solution was added, and the reaction was arrested. The color developed, being proportional to the amount of biomarker bound in the initial step, allowed measuring its intensity using spectrophotometry (450/620 nm, ELISA processor). A calibration curve was plotted by regression analysis, and the optical density of the sample was used to estimate the concentration of the biomarkers. The concentrations were expressed as biomarker per 30 s (pg/30 s). During the laboratory analysis the samples were blind to the lab technician.

### 2.7. Statistical Analysis

Continuous variables were presented with mean and standard deviation, whereas for categorical variables frequencies and percentages were used. The assumption of normal distribution was investigated for all the variables using Shapiro-Wilk test. Thus, parametric, and non-parametric tests were used. Independent samples *t*-test and Mann Whiney U test were used to compare the changes in clinical parameters: mean PPD, mean PAL, mean REC, %BoP-yes, and the protein parameters (RANKL, OPG) between the two groups (control, laser). Paired *t*-test and Wilcoxon signed rank test were used to compare the clinical and protein parameters between baseline and six months in each group separately. Statistical analysis was performed using STATA 13 (StataCorp LP, College Station, TX, USA). The statistical significance level was set at *p*-value ≤ 0.05.

## 3. Results

### 3.1. Demographic Characteristics of the Participants

**Figure 3:**
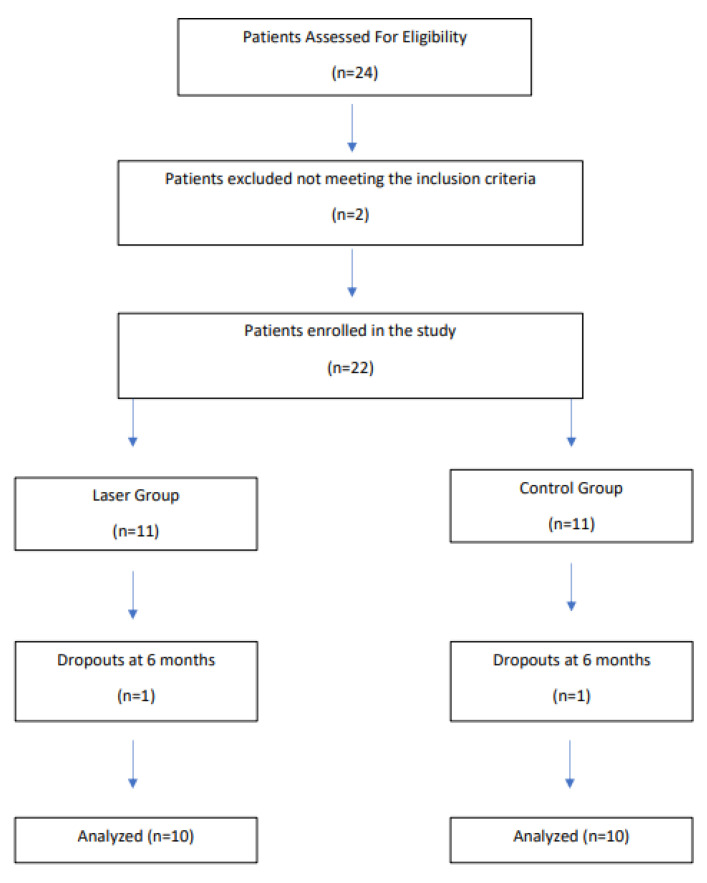
Patient Inclusion.

Twenty subjects with 20 implants completed the study, and two implants were lost to follow-up due to the inability to re-examination because of the pandemic (Figure 3). All patients had been previously treated for periodontal disease and were following supportive periodontal therapy. In addition, the included participants were no smokers or smoked < 5 cigarettes/day. All implants included in the study presented a modified surface. The mean age of the subjects was 58.10 (SD, 8.54) in the laser group and 60.28 (SD, 6.34) in the control group, with no group difference (*p* = 0.575). Statistical analysis also failed to demonstrate study group gender differences (*p* = 1.0) (Table 1).

### 3.2. Clinical Parameters

#### 3.2.1. Baseline and Six-Month Clinical Parameters

The comparison of the baseline clinical parameters among groups revealed no differences in none of the examined clinical parameters (Table 2). At the six months, the clinical values were similar for both groups in terms of FMPS, PPD, PAL, and REC; however, the percentage of BoP-positive sites was higher in the control group. (69.05 ± 40,17 vs. 15 ± 18, 34), *p* = 0.002) (Table 2). The inter-group comparisons for the clinical changes between baseline and six months showed no difference in the FMPS, PPD, PAL, or REC changes among the two groups. A higher reduction in the BoP-positive sites was recorded for the laser group (19.05 ± 33.9 vs. 55.00 ± 30.48, *p* = 0.037) (Table 3).

#### 3.2.2. Biomarkers Assessment

The results of ELISA analysis revealed that only 29 out of the evaluated 40 PICF samples were above the minimum detection limit of the sRANKL assay kit, and 28/40 PICF samples revealed OPG amounts above the minimum detection limit of the assay kit. The baseline and six-month comparisons of sRANKL and OPG revealed no statistically significant differences between the two groups (Table 4). Due to the limited number of samples, all values below detection limits were considered missing and were excluded from the analysis. Thus, the comparison among the changes in biomarker values was available for a lower number of participants. The results revealed no difference in the changes in sRANKL and OPG levels among the two groups after six months (Table 5).

## 4. Discussion

Peri-implantitis is considered a microbial inflammatory disease, rather complicated in nature, affecting the implant surrounding tissues. Peri-implantitis is characterized by gradual bone loss analogous to periodontitis [25]. Though treatment protocols have been established in the latter, in peri-implantitis, most treatment protocols are still considered empirical, and not a uniformly acceptable treatment strategy has been proposed. Several decontamination methods combined with surgical or non-surgical processes have been investigated. Most of them provide limited improvement in terms of inflammation resolution and halt of disease progression. In the current investigation, clinical and biomarker values were compared to a control group six months after treatment.

The study results indicated statistically significant improvements in the clinical parameters during the 6-month observation period for both groups. More specifically, PPD, PAL, and REC were improved in both groups, with no differences in the between-groups comparisons. However, a greater reduction in the BoP-positive sites was noted for the combined laser irradiation therapy. Most studies on surgical peri-implantitis treatment with the conjunctional application of lasers conclude that slight superiority occurs compared to other treatments [26]. Long-term studies although reporting a higher reduction in BoP levels in the short term suggest that in the long-term clinical improvements are not influenced by the initial method of surface decontamination [27]. In addition, the consensus report of the American Academy of Periodontology (AAP) regarding the efficacy of Lasers as adjuncts in surgical peri-implantitis treatment, reported a weighted mean difference (WMD) in terms of PPD, PAL, BoP, and PI of 0.45 mm (*p* = 0.11), 0.22 mm (*p* = 0.56), 7.26% (*p* = 0.76) and −0.09 (*p* = 0.84), respectively. These values do not indicate any superiority of the Laser compared to conventional mechanical treatment. However, up to this point, the number of RCTs on the topic remains limited. The wide range of lasers along with different properties and settings (i.e., wavelength, power, waveform, pulse duration, energy/pulse, the density of the energy, duration of the exposure, angulation of the energy toward the targeted tissue, peak power of the pulse, and the properties of tissue), as applied in various studies could strongly influence the treatment [28].

To the best of our knowledge, this is the first publication to report on the effect of combined irradiation of Er: YAG and Nd: YAG lasers on the implant surface and peri-implant tissues in the surgical treatment of peri-implantitis defects. Moreover, there is a limited number of studies using laser irradiation in conjunction with surgical peri-implantitis therapy. More specifically, in the study by Papadopoulos et al, a diode laser was used as an adjunct to access flap, yielding similar clinical improvements for all clinical parameters in both groups six months after treatment, concluding that the adjunctive use of a laser is not beneficial in peri-implantitis therapy [29]. Furthermore, Albaker et al., using photodynamic therapy in combination with a diode laser for the treatment of peri-implant defects, found an equal (1.3 mm) reduction in PPDs for both groups suggesting that a single application of aPDT as an adjunct to access flap does not provide additional benefit in improving clinical and radiographic peri-implant parameters in peri-implantitis [30].

In terms of BoP changes, significant reductions were observed in both groups between baseline and six months (C: 88.09 ± 20.8), 69.05 ± 40.17, *p* = 0.188, T: 70.00 ± 25.82,15 ± 18.34, *p* < 0.003). However, the reduction in BoP-positive sites was greater in the laser group (Mean change 19.05 ± 33.92 vs. 55.00 ± 30.48, *p* = 0.037.) This is in accordance with an early study using Er: YAG lasers in the non-surgical therapy of peri-implantitis, where a significantly higher reduction in BoP was noticed 3, 6 and 12 months after treatment [31]. Most studies reporting on the use of Er: YAG laser in non-surgical peri-implantitis treatment did not find significant differences regarding BoP values between the laser and the control group [22,23]. Nevertheless, findings of studies on surgical peri-implantitis therapy involving Er: YAG laser indicated similar changes in BoP values between the test and control groups in the short and long term [27,32] a finding which is not consistent with ours. To interpret these incompatible findings, several factors need to be analyzed. First, it has to be emphasized that previous in vitro studies have pointed out a high bactericidal potential of Er: YAG Laser on common dental implant surfaces, even though a complete bacterial reduction following laser irradiation could not be observed [33,34]. Furthermore, several studies have reported on the removal of plaque biofilms and calculus from both smooth and rough titanium surfaces, including the area of screw threads, as well as on antimicrobial effects against periodontopathic bacteria and the removal of lipopolysaccharides by Er: YAG laser radiation [35].

Combining the two types of lasers in periodontitis has shown promising results for clinical attachment gain and pocket depth reduction in the short term (3 months) [36,37]. Moreover, the combined treatment has shown favorable microbiological responses with a higher reduction in microbial levels for the combined groups [14]. However, Nd: YAG laser has scarcely been investigated in the case of peri-implantitis. Abduljabbar et al. observed that Nd: YAG laser-assisted non-surgical mechanical debridement may lead to a more pronounced improvement of peri-implant soft tissue inflammatory parameters than mechanical debridement alone in the short-term yielding a higher reduction in BoP values at three months and promote healing [38].

Biomarkers have been widely used in several medical fields to aid the diagnosis, prognosis, and disease monitoring. The RANKL/OPG system has been recognized as a critical bone metabolism regulator. RANKL is the primary factor responsible for osteoclast cell differentiation [18]. In vitro binding of RANK with its cognate RANKL results in osteoclastogenesis by monocyte/macrophage progenitor differentiation to osteoclasts and the activation of mature osteoclasts [39]. OPG, on the other hand, functions as a soluble decoy receptor for RANKL and competes with RANK for RANKL binding. Therefore, OPG is an effective inhibitor of osteoclast maturation and osteoclast activation in vitro and in vivo [40]. An increased RANKL/OPG ratio has been reported in periodontitis sites compared with gingivitis/healthy sites. In addition, recent studies suggest a correlation between sRANKL, OPG, and their ratio with peri-implantitis and clinical markers such as BoP and PPD [21,41].

In the current study, the effect of combined laser application in the surgical management of peri-implantitis in RANKL and OPG was investigated for six months. The results showed that none of the treatment modalities was found superior in modifying the behavior of RANKL and OPG at the baseline or six months, though a trend towards a higher reduction in sRANKL was noticed in the combined laser irradiation group (3.97 vs. 1.52 pg/30 s). However, in the ELISA analysis, 32% of the samples were below the detection limit for both proteins, which means that a potential difference in the biomarker response between the two groups may be concealed due to missing values. Arikan and colleagues, in an effort to correlate sRANKL and OPG levels with clinical values, detected sRANKL in only 12% of the specimens and suggested that the pooling of samples from each implant would have yielded higher detection values. In addition, they assumed that excluding samples that were below the detection limit would produce bias, so they included them in the analysis calculated as zero values [42]. In contrast, other studies used only one paper strip to detect several proteins, yielding varying results [19,21]. These differences may possibly be explained by the differences in the detection methodology, the sensitivity of the assay, and the detectability rates used in the reported studies. In the current study, samples that were below the detection limit were considered missing values and were excluded from the analysis. Also, in the present study, only implants diagnosed with peri-implantitis were included compared to earlier studies using healthy controls. The inflammatory status of the tissues may have affected the PICF sample collection, thus influencing the ELISA results.

When evaluating the biomarkers, we found an increase in sRANKL was noticed for the control group [3.65 ± 2.94–11.27 ± 15.08) pg/30 s], while a slight reduction was found in the laser group [6.63 ± 5.42–3.35 ± 3.22) pg/30 s], though the changes could not be assessed statistically. Those results should be interpreted with caution as they do not reflect the treatment effects. A more accurate way to determine the treatment effects would be the RANKL/OPG ratio, which could not be analyzed in the current study due to the reduced number of samples and missing values. Reports on the effects of treatment on RANKL/OPG are scarce in the literature. In the case of peri-implantitis, only one study utilizing surgical peri-implantitis therapy by means of titanium curettes and chlorhexidine has tested the effects on the RANKL/OPG system [43]. The study results indicated an increase in the OPG/RANKL ratio three months after treatment. However, no change in the OPG levels was found. Instead, a reduction in the RANKL values was more evident. The difference between that study and the current might lie in the evaluation time points (6 vs. 3 months). A more recent study estimated the diagnostic accuracy of the biomarkers mentioned above in the case of peri-implantitis. The results indicated that the values of sRANKL and OPG have a higher diagnostic accuracy individually rather than their ratio [20]. Belibasakis et al., in a review, concluded that the RANKL/OPG ratio maybe not be reduced after periodontal treatment, indicating that it may not act as a marker for a stable periodontium rather than an indicator of periodontal disease history. Whether this is the case in peri-implantitis, remains to be investigated [17].

Prior to conclusions some limitations of the study were the relatively small number of partcipants. Though the sample size calculation indicated sufficient power of the sample, it is our belief that a higher number of participants would elucidate more the exact role of the biomarkers. In addition, the number of samples that were below the detection limit did not allow for a RANKL/OPG ratio calculation to assess the immunomodulatory effects of the treatment as described earlier.

## 5. Conclusions

Within the limits of the current study, it can be concluded that:

- The combined Nd: YAG—Er: YAG laser surgical therapy of peri-implantitis seems to lead to more significant improvements in bleeding on probing six months after treatment compared to conventional mechanical decontamination of the implant surface.
- None of the methods was found superior in modifying the behavior of bone loss biomarkers (RANKL, OPG) six months after treatment.

## 6. Future Research Recommendations

The current study indicated a potential for the combined laser application to reduce inflammation of peri-implant tissues. Since this is the first study using a combined laser irradiation therapy in the treatment of peri-implantitis, future prospective studies including a higher number of participants should be planned to evaluate the full potential of the treatment. In addition, long-term follow-up studies should investigate the effects of the combined treatment in a more objective way.

The exact role of RANKL and OPG in peri-implantitis needs to be further investigated by cohort studies with a large number of participants, and the effect of peri-implantitis treatment on the later biomarkers should be addressed in order to evaluate the diagnostic and prognostic accuracy of both proteins.

## Figures and Tables

**Table 1:** Baseline characteristics of the study patients.

	Control (n = 10)	Laser (n = 10)	*p*-Value
Age in years, mean (SD)	60.28 ± 6.34	58.10 ± 8.54	0.575 ^a^
Females/Males (n)	4/6	5/5	1.000 ^b^

SD: Standard deviation; ^a^: independent sample to test; ^b^: Chi-squared test.

**Table 2:** Baseline and 6-month clinical values.

	Baseline		
	Control (n = 10) Mean ± SD	Test (n = 10) Mean ± SD	*p*-Value
FMPS %	9.89 ± 1.25	10.3 ± 1.27	0.324 a
Mean PPD (mm)	9.32 ± 1.19	7.85 ± 1.05	0.09 a
Mean PAL (mm)	9.57 ± 1.13	8.20 ±1.39	0.24 a
Mean REC (mm)	0.25 ± 0.38	0.35 ± 0.57	0.348 b
Mean % BoP	88.09 ± 20.89	70.00 ± 25.82	0.146 a
	**6 months**		
	**Control (n = 10)** Mean ± SD	**Test (n = 10)** Mean ± SD	***p*-Value**
FMPS %	7.9 ± 1.21	8.21 ± 1.39	0.521 a
Mean PPD (mm)	6.71 ± 1.38	5.70 ± 1.05	0.053 a
Mean PAL (mm)	7.42 ± 1.13	6.30 ± 1.39	0.45 a
Mean REC (mm)	0.71 ± 0.48	0.60 ± 0.69	0.358 a
Mean % BoP	69.05 ± 40.17	15 ± 18.34	0.002 a *

PD: Probing Depth; CAL: Clinical attachment level; BOP: Bleeding on Probing; SD: Standard Deviation; ^a^ Independent samples *t*-test; ^b^ Mann-Whitney U test; * Statistically significant at 0.05 level.

**Table 3:** Comparisons of the changes in clinical values between groups.

	Control (n = 10) Mean ± SD	Test (n = 10) Mean ± SD	*p*-Value
Mean Change in FMPS% (Baseline–6 months)	1.99 ± 1.21	2.09 ± 1.3	0.234 ^a^
Mean change in PPD (Baseline–6 months)	2.60 ± 1.70	1.95 ± 0.95	0.163 ^a^
Mean change in PAL (Baseline–6 months)	2.14 ± 1.46	1.90 ± 1.26	0.361 ^a^
Mean change in REC (Baseline–6 months)	−0.46 ± 0.47	−0.25 ± 0.92	0.291 ^a^
Mean change in % BoP (Baseline–6 months)	19.05 ± 33.92	55.00 ± 30.48	0.037 ^a,^*

PD: Probing Depth; CAL: Clinical attachment level; BOP: Bleeding on Probing; SD: Standard Deviation; ^a^ Independent samples *t*-test; * Statistically significant at 0.05 level.

**Table 4:** Baseline and six-month values of OPG and sRANKL in the two groups.

	Control Mean ± SD	Test Mean ± SD	*p*-Value
Mean RANKL baseline (pg/30 s)	3.65 ± 2.94	11.27 ± 15.08	0.250 ^a^
Mean OPG baseline (pg/30 s)	33.36 ± 12.00	25.22 ± 17.34	0.345 ^a^
Mean RANKL 6 months (pg/30 s)	6.63 ± 5.42	3.35 ± 3.22	0.233 ^a^
Mean OPG 6 months (pg/30 s)	31.05 ± 29.24	20.18 ± 20.16	0.529 ^a^

^a^ Independent samples *t*-test; SD: Standard Deviation.

**Table 5:** Comparison of the changes in biomarker levels between the two groups.

	Control Mean ± SD	Test Mean ± SD	*p*-Value
Mean change in RANKL (pg/30 s) (Baseline—6 months) (n = 8)	1.52 ± 1.03	−3.97 ± 2.94	0.123 ^a^
Mean change in OPG (pg/30 s) (Baseline—6 months) (n = 9)	−3.16 ± 1.47	−4.22 ± 1.03	0.143 ^a^

^a^ Independent samples *t*-test.

## Data Availability

Data available upon request to the author.
